# Sub-minimum inhibitory concentrations (sub-MICs) of colistin on *Acinetobacter baumannii* biofilm formation potency, adherence, and invasion to epithelial host cells: an experimental study in an Iranian children’s referral hospital

**DOI:** 10.1128/spectrum.02523-23

**Published:** 2024-01-17

**Authors:** Neda Yousefi Nojookambari, Gita Eslami, Mehrzad Sadredinamin, Maryam Vaezjalali, Bahram Nikmanesh, Razieh Dehbanipour, Sajjad Yazdansetad, Zohreh Ghalavand

**Affiliations:** 1 Department of Microbiology, School of Medicine, Shahid Beheshti University of Medical Sciences, Tehran, Iran; 2 Department of Medical Laboratory Sciences, School of Allied Medical Sciences, Tehran University of Medical Sciences, Tehran, Iran; 3 Department of Microbiology, School of Medicine, Yasuj University of Medical Sciences, Yasuj, Iran; 4 Infectious Diseases Research Center, Golestan University of Medical Sciences, Gorgan, Iran; NHLS Tygerberg/Stellenbosch, Cape Town, Western Cape, South Africa

**Keywords:** colistin, virulence, biofilm, children, epithelial Cells, sub-MIC

## Abstract

Here, we described the efficacy of colistin sub-minimum inhibitory concentrations (sub-MICs) on biofilm-forming activity, host epithelial cell adherence, and invasion capacity of *Acinetobacter baumannii* strains collected from children admitted to the Children’s Medical Center Hospital. Biofilm formation potency of *A. baumannii* clinical isolates was measured using a 96-well microtiter plate assay. Distribution of biofilm-related genes, including *bap*, *abaI*, *ompA*, *csuE*, and *bla*_PER-1_, was detected by PCR. The mRNA expression level of *ompA* and *csuE* was measured by qPCR in the presence of ¼ and ½ MICs of colistin. *A. baumannii* adhesion and invasion to eukaryotic host cells were phenotypically assayed at sub-MICs of colistin. Eighty percent (56/70) and 35.7% (25/70) of *A. baumannii* isolates were multidrug-resistant (MDR) and extensively drug-resistant (XDR) phenotypes, respectively. The strong, moderate, and weak biofilm producers of *A. baumannii* were 37.1% (26/70), 32.8%, (23/70), and 22.8% (16/70), respectively. The frequencies of biofilm-associated genes were 100% for *abaI*, *ompA*, and *csuE*, followed by 22.8% (16/70) and 24.3% (17/70) for *bap* and *bla*_PER-1_, respectively. The downregulation of *csuE* and *ompA* expression levels was observed in the sub-MIC of colistin. *In vitro* cell culture study showed a decreased capability of *A. baumannii* to adhere to the human epithelial cells at sub-inhibitory doses of colistin; however, none of the isolates could invade HEp-2 cells. Our study showed that the genes encoding biofilm-associated proteins undergo downregulation in expression levels after exposure to sub-MICs of colistin in *A. baumannii*. Longitudinal *in vivo* studies are needed to fully understand the clinical aspects of pathogenicity mechanisms and evolutionary dynamics of drug resistance.

**IMPORTANCE:**

Since the toxicity of colistin is dose dependent, there is a focus on strategies that reduce the dose while maintaining the therapeutic effect of the drug. Our findings about sub-inhibitory doses of colistin provide a novel insight into the logical use of colistin to treat and control *Acinetobacter baumannii*-related infections in clinical practice.

## INTRODUCTION

*Acinetobacter baumannii* is listed as a global priority pathogen on the WHO’s list of antibiotic-resistant bacteria presenting the most serious threat to human health (1). It belongs to the ESKAPE pathogen (*Enterococcus faecium*, *Staphylococcus aureus*, *Klebsiella pneumoniae*, *Acinetobacter baumannii*, *Pseudomonas aeruginosa*, and *Enterobacter* species), a group of pathogens with a high rate of antibiotic resistance and is mainly responsible for nosocomial infections (2).

Over the past few decades, *A. baumannii* has become a challenging organism, especially in pediatrics and people with compromised immune systems due to hospital-derived infections and biofilm-associated complications such as catheter-related bloodstream/urinary tract infections and ventilator-associated pneumonia (3).

It has been shown that *A. baumannii* can attach to host cells while some have invasive potential (4). Adherence to host cells is an initial step for colonization and the formation of a highly structured microbial community (5). The interactions of host-pathogen trigger cell signaling to establish multiple infections (6).

Although *A. baumannii* is reported to have limited virulence potential, the incidence of multiple drug resistance, strong capability to form biofilm on biotic and abiotic surfaces, and adherence and invasion to host cells increase their survival and colonization in hospital environments as well as patient’s indwelling devices and limit our ability to treat and eliminate the infections (7, 8).

Today, outbreaks of *A. baumannii* are increasing in occurrence and severity due to its multidrug- and extensively drug-resistant (MDR and XDR) nature (9).

Now, polymyxins and tigecycline are the “last-resort” effective antibiotics for the treatment of MDR *A. baumannii* infections (10). Currently, the prevalence of resistance to colistin (polymyxin class) is relatively low; however, rapid global resistance toward colistin has emerged following its resurgence (11). The resistance rates of 20% and 50% to polymyxins and tigecycline, respectively, have been reported in previous studies (12, 13). Nevertheless, the lack of therapeutic success with the last-resort antibiotics against MDR, XDR, and biofilm-forming *A. baumannii* leads to an increase in mortality and morbidity, especially in children (14).

Antibiotics can act successfully in the inhibition of the growth of pathogens when their concentration is higher than the minimum inhibitory concentration (MIC) between consecutive doses (15). However, the prescribed dosage of antibiotics is usually diminished in target tissues and becomes a concentration lower than the MIC in several treatment regimens, resulting in pathogens being weakly inhibited. Therefore, the antibiotic-susceptible dynamic clones continue to grow at sub-MICs despite the hazards of antibiotic presence (16).

It has been reported that the exposure of bacteria to sub-inhibitory doses of antibiotics may induce metabolic stress on microorganisms living in biofilm communities, enhance the capacity of bacteria to resist higher doses of antibiotics, promote the synthesis of enzymes and toxins, change genetic levels and bacterial ultrastructure, encourage prophage, induce adhesion properties and virulence factors, and stimulate biofilm formation by modulating the expression levels of the biofilm-related genes (17, 18). The mechanisms differ across species and drug classes and are of great interest for ongoing research. Antibiotic-induced biofilm formation has been reported in previous studies (19–21). However, little information exists on the molecular mechanisms behind the sub-minimum inhibitory concentrations (sub-MICs) of antibiotic-induced biofilm formation.

Some previous studies described that the exposure of *A. baumannii* to sub-MICs of colistin may launch transcriptional and post-transcriptional modifications in the biofilm-dwelling microorganisms, resulting in biofilm-associated drug resistance in *A. baumannii* (22, 23). The biofilm lifestyle allows *A. baumannii* to withstand hostile environmental conditions and makes it capable of causing a broad range of chronic infections (24).

The antimicrobial therapy of infections caused by *A. baumannii* in pediatric patients is challenging and requires an effective approach to improving antimicrobial use, with a view to improve clinical outcomes and minimize adverse events such as the development of antimicrobial resistance. Here, we aimed to investigate the sub-MICs of colistin on biofilm formation potency, host cell adherence, and invasion capacity of *A. baumannii* strains collected from children admitted to the Children’s Medical Center Hospital.

## MATERIALS AND METHODS

### Clinical specimens, bacterial isolates, and characterization

A total of 70 non-duplicate *A. baumannii* isolates were recovered from the blood, wound, throat, eye, bronchoalveolar lavage (BAL), cerebrospinal fluid (CSF), esophagus, urine, sputum, drain discharge, tracheal tube, dialysis fluid, central venous line, intravenous (IV) catheter, and nasopharyngeal secretions of pediatric patients, aged between 4 days and 14 years, hospitalized in different divisions of Children’s Medical Center in a period of 31 months from April 2019 to December 2021. Children’s Medical Center is the oldest (since 1968) and the largest referral pediatric hospital and it is known as a center of excellence in pediatrics in Iran. Standard microbiological methods, including Gram staining, oxidase, catalase, oxidation-fermentation (OF), methyl red, Voges-Proskauer, TSI, H_2_S production, indole formation, motility, and growth at 44°C were used to phenotypically identify *A. baumannii* isolates. As well as, the isolates were established by PCR amplification of *bla*_OXA-51-like_ and partial RNA polymerase β-subunit (*rpoB*) inherent genes using the two sets of forward and reverse primers, as described in Table 1. *A. baumannii* pure cultures were stored frozen at -80°C in tryptic soy broth (TSB) supplemented with glycerol 20% (vol/vol) for further use.

**TABLE 1:** Gene target, primer sequences, product sizes, and annealing temperatures for identification of *A. baumannii* isolates and characterization of biofilm-encoding genes

Target gene	Primer sequence (5´−3´)	Product size (bp)	Annealing temperature (°C)	Reference
*bla*_OXA-51-like_	F: TAA TGC TTT GAT CGG CCT TG R: TGG ATT GCA CTT CAT CTT GG	353	53	(25)
*rpoB*	F: TAY CGY AAA GAY TTG AAA GAA G R: CMA CAC CYT TGT TMC CRT GA	543	50	(26)
*ompA*	F: CTGGTGTTGGTGCTTTCTGG R: GTG TGA CCT TCG ATA CGT GC	352	49	(27)
*csuE*	F: AGA CAT GAG TAG CTT TAC G R: CTT CCC CAT CGG TCA TTC	516	52	(28)
*bla*_PER-1_	F: GCA ACT GCT GCA ATA CTC GG R: ATG TGC GAC CAC AGT ACC AG	340	58	(27)
*abaI*	F: CGC TAC AGG GTA TTT GTT GA R: TCG TAA TGA GTT GTT TTG CG	370	46	(27)
*bap*	F: ATA ACT CGG CTG TTT ACG G R: ACT GAT GGT GTT GGA AGT G	358	49	(27)

### Antimicrobial susceptibility testing

The Kirby-Bauer disk diffusion susceptibility test was used to determine the sensitivity or resistance of *A. baumannii* clinical isolates to various antibiotics according to Clinical and Laboratory Standards Institute (CLSI) guidelines (29) and breakpoint zone size interpretations for including ceftazidime (30 µg), cefepime (30 µg), ceftriaxone (30 µg), cefotaxime (30 µg), piperacillin-tazobactam (100/10 µg), ampicillin-sulbactam (10/10 µg), imipenem (10 µg), meropenem (10 µg), gentamicin (10 µg), tobramycin (10 µg), amikacin (30 µg), ciprofloxacin (5 µg), levofloxacin (5 µg), trimethoprim-sulfamethoxazole (1.25/23.75 µg), doxycycline (30 µg), tetracycline (30 µg), minocycline (30 µg), and tigecycline (15 µg). All antibiotic discs were purchased from MAST Group Ltd., Merseyside, UK. *Pseudomonas aeruginosa* ATCC 27853 and *Escherichia coli* ATCC 35218 were used as the standard strains.

MIC of colistin (Colistin sulfate salt powder, Sigma-Aldrich, St. Louis, MO, USA) against 70 *A*. *baumannii* isolates was determined using broth microdilution technique according to CLSI (29) guidelines. *A. baumannii* ATCC 19606 and *E. coli* ATCC 25922 were used as the standard strains for MIC value.

MDR, XDR, and PDR strains of *A. baumannii* were defined as previously described by Magiorakos et al. (30).

### Biofilm formation assay

Biofilm formation activity of *A. baumannii* clinical isolates and *A. baumannii* ATCC 19606 was assayed in the absence and presence of sub-inhibitory concentrations (¼ and ½ MICs) of colistin in the 96-well optically clear flat-bottom plate using a previously described method with some modifications (31). Briefly, bacterial isolates were aerobically cultured in the TSB, with shaking at 200 rpm, at 37°C for 18 hours. Bacterial cell suspension was adjusted to a 0.5 McFarland standard [~1 × 10^8^ colony forming units (CFU) mL^−1^], then added to a 96-well plate containing fresh TSB to obtain a concentration of ~1 × 10^6^ CFU mL^−1^. Each bacterial isolate was added to the plate in triplicate. TSB without bacterial cell suspension was set up as a negative control. After incubation at 37°C for 24 hours, the contents of the wells were discarded, and adherent cells were washed three times with phosphate-buffered saline (PBS, pH 7.4) to remove the planktonic cells. Then, biofilms were fixed with 250 µL methanol 99% (vol/vol), and wells were stained with crystal violet 0.1% (wt/vol) for 20 min at room temperature (RT). Crystal violet was dissolved using acetic acid 33% (vol/vol) and incubated for 5 min at RT with shaking at 125 rpm. The absorbance (optical density; OD) of the eluted solvent was measured at 570 nm using Synergy HTX Multi-Mode Microplate Reader (BioTek, US). Biofilm formation of the isolates was scored as follows: if ODs ≤ ODc, the bacteria were non-adherent; if ODc <ODs ≤ 2 × ODc, the bacteria were weakly adherent; if 2 × ODc < ODs≤4 × ODc, the bacteria were moderately adherent; if 4 × ODc < ODs, the bacteria were strongly adherent. ODs are defined for the clinical strains and ODc for the negative control.

### Distribution of biofilm-associated genes

Genomic DNA of the fresh overnight cultures of bacterial cells was extracted by the HiPurA Bacterial Genomic DNA Purification Kit (HiMedia, India).

The distribution of biofilm-related genes, including *bap*, *abaI*, *ompA*, *csuE*, and *bla*_PER-1_, was detected by conventional PCR. The sequences of primers (Copenhagen, Denmark), PCR product sizes, and annealing temperatures for these target genes are described in Table 1.

PCR amplification of the genes was done in a final volume of 25 µL reaction mixture containing 12.5 µL of 2X *Taq* DNA Polymerase Master Mix RED (Ampliqon, Denmark), ~25 ng of DNA template, and 10 pmol of each forward and reverse primers.

PCR was performed in a thermocycler machine Bio Intellectica Gene-Explorer (Bio Intellectica, Canada) under thermal cycling conditions: initial denaturation at 94°C for 4 min, followed by 30 cycles of denaturation at 94°C for 45 s, annealing at different temperatures (see Table 1) for 45 s, extension at 72°C for 45 s, and final extension at 72°C for 5 min. PCR products were separated in a 1.5% agarose gel by electrophoresis and visualized by DNA Safe staining under ultraviolet (UV) light.

### Sequencing and bioinformatics analysis

The amplicons were sequenced using an ABI 3730XL DNA Analyzer (Applied Biosystem Inc., Foster City, CA) using Sanger (dideoxy chain termination) technology. Nucleotide sequences were analyzed using Chromas v.2.6.6 (http://www.technelysium.com.au) and BlastN and BlastX BlastNetwork algorithms available in the NCBI database (https://blast.ncbi.nlm.nih.gov/Blast.cgi). The nucleotide sequences were translated into amino acids and protein coding sequences were annotated *via* EMBOSS Transeq tool (https://www.ebi.ac.uk/Tools/st/emboss_transeq/).

### Cell culture

Human laryngeal epithelial (HEp-2) cells were cultured in a T-25 flask containing high glucose Dulbecco’s Modified Eagle Medium (DMEM) supplemented with 2 mM L-glutamine, 1% Penicillin-Streptomycin (PenStrep), and 10% (vol/vol) heat-inactivated fetal bovine serum (Gibco, Brazil), then incubated at 37°C in a humidified atmosphere with 5% CO_2_. At approximately 90% confluency, the cells were harvested using trypsin-EDTA, and new stock cultures were prepared by seeding 2 × 10^6^ cells/mL onto the DMEM with supplements. Confluent growth was obtained in 100 mm diameter in 24-well plates for further steps.

### Bacterial adherence and invasion

Bacterial adherence and invasion to the eukaryotic cells were assayed as previously described with several modifications (32). HEp-2 cells were used to determine the adherence and invasion abilities of six selected isolates of *A. baumannii* in the absence and presence of sub-MICs of colistin. The characteristics of the selected isolates were as follows: All the isolates were biofilm producers, as well as which had at least five genes involved in biofilm formation and adhesion to biotic and abiotic surfaces. Five isolates were considered strongly adherent, and the other was considered weakly adherent.

#### Adherence assay

HEp-2 cells were seeded in a 24-well plate at a density of about 2 × 10^5^ cells per well. Then, allowed to adhere for 24 hours at 37°C with 5% CO_2_, in 1 mL of drug-free DMEM (dfDMEM) with 10% FBS. *A. baumannii* isolates were aerobically cultured overnight on Mueller-Hinton agar plates at 37°C, then suspended in plain DMEM containing 0 MIC, ¼ MIC, and ½ MIC of colistin. An aliquot of 1 mL of bacterial cell suspension of a density corresponding to approximately 1.5 × 10^8^ CFU/mL was added to the HEp-2 cells. The density of the suspension was approximated photometrically. Prior to co-culture with *A. baumannii*, the monolayer HEp-2 cells were washed three times with sterile phosphate-buffered saline (PBS). The cells were then co-cultured at a multiplicity of infection of 100 bacteria per cell (MOI 1:100) at 37°C under 5% CO_2_ for 1 hour. At the end of the exposure time, eukaryotic monolayers were washed three times with sterile PBS and harvested after trypsinized HEp-2 cells by adding sterile PBS to each well; the total bacterial count was estimated by the growth of serial dilutions (1:10, 1:100, 1:1000) of the bacterial suspension on Mueller–Hinton agar based on CFU after 24 hours of incubation at 37°C. The adherence rate of bacterial strains to the HEp-2 cells in three conditions, that is, colistin-free (0 MIC), ¼ MIC, and ½ MIC of colistin was calculated based on the following formula:


$$Number\;of\;colonies\;adhered\;to\;the\;HEp-2\;cells=colony\;count\times dilution\;factor\times \left(\frac{\text{volume of microbial solution inoculated to MHA}}{\text{final volume of each well containing lysate}}\right)$$

Moreover, the viability of host cells throughout the incubation period was authenticated by trypan blue staining.

#### Invasion assay

The HEp-2 cells grown in 24-well plates were infected with *A. baumannii* for up to 5 hours at an infection of 100 bacteria per cell (MOI 1:100). Culture media were discarded and the monolayer HEp-2 cells were washed three times with sterile PBS. Following incubation, the wells were treated with 300 µg/mL of gentamicin or 300 µg/mL of colistin dissolved in dfDMEM and incubated for 1 hour to kill extracellular bacteria. Colistin was used due to the resistance of the isolates to gentamicin. The HEp-2 cells were washed three times with PBS and lysed with 0.1% Triton X-100 at 37°C for 20 min. Serial dilutions from each well were plated on Mueller-Hinton agar and colonies were counted 24 h after incubation.

### RNA extraction and quantitative real-time PCR

Total RNA of bacterial isolates following 18 hours of incubation in the absence and presence of sub-inhibitory concentrations (¼ and ½ MICs) of colistin was prepared from BioFact Total RNA Prep column type kit (BioFACT, Korea). The quantification of RNA was assayed using a DeNovix DS-11 Spectrophotometer (DeNovix, US).

Two micrograms of DNase-treated RNA was reverse transcribed using the mixture of random 6-mer primers of BioFact RT Series 1st strand cDNA synthesis kit (BioFACT, Daejeon, Korea) according to the manufacturer’s instructions. The cDNA was amplified using the SYBR Green 2X Real-Time PCR Master Mix (BioFACT, Korea) by consensus primers for detecting *rpoB*, *csuE*, and *ompA*. The *rpoB* was used as an internal control for the quantification of target genes. The primer sequences are listed in Table 2. The reaction was done in a Corbett Research Rotor-Gene 6000 Real-Time PCR machine (Corbett Life Science, Australia) as follows: initial denaturation at 95°C for 15 min, followed by 40 cycles of denaturation at 95°C for 30 s, annealing at 57°C (for all three target genes) for 30 s, extension at 72°C for 30 s, and final extension at 72°C for 5 min. Fold changes in the mRNA expression were calculated according to the C_t_ method (2^−ΔΔCt^) using *rpoB* as the normalized reference gene. The fold changes ≥ 2 and ≤0.5 were considered significant.

**TABLE 2:** Gene target, primer sequences, product sizes, and annealing temperatures for relative expression assay of genes involved in biofilm formation in clinical isolates of *A. baumannii*

Target gene	Primer sequence (5´−3´)	Amplicon length (bp)	Annealing temperature (°C)	Reference
*rpoB*	F: GAG TCT AAT GGC GGT GGT TC R: ATT GCT TCA TCT GCT GGT TG	110	57	(33)
*csuE*	F: TCA GAC CGG AGA AAA ACT TAA CG R: GCC GGA AGC CGT ATG TAG AA	150	57	(34)
*ompA*	F: TCT TGG TGG TCA CTT GAA GC R: ACT CTT GTG GTT GTG GAG CA	86	57	(35)

### Statistical analysis

All data were subjected to analysis using the IBM SPSS Statistics 2019 v26.0 (IBM Corp., Armonk, NY, USA) tool. Spearman’s rank correlation and Fisher’s exact test were used to analyze the correlation between biofilm formation and antibiotic resistance/susceptibility. Spearman’s test was also used for the correlation of gene expression and biofilm formation. Two-tailed *P* value ≤ 0.050 was considered statistically significant. GraphPad Prism software version 9.0.0 (GraphPad Software Inc., San Diego, CA, USA) and one-way ANOVA were used for adherence assay and qPCR data analysis.

## RESULTS

### Demographic characteristics and laboratory findings

A total of 70 non-replicate *A. baumannii* isolates were obtained from pediatric patients. 49 out of 70 (70%) and 21 out of 70 (30%) isolates of *A. baumannii* were collected from male and female pediatric patients, respectively. 61.4% of patients were infants with an age of ≤1 year and 10% were aged ≥10 years. Neonatal, pediatric, and emergency intensive care units (NICU, PICU, and EICU) had the biggest share of total isolates (69.6%), followed by the other wards (30.4%). The distribution of isolates from the clinical origin was, respectively, as follows: Blood (30%), BAL (22.9%), tracheal tube (14.3%), throat (4.3%), wound (2.9%), eye (2.9%), CSF (2.9%), urine (2.9%), dialysis fluid (2.9%), sputum (1.4%), and others (12.9%).

The demographic data, clinical characteristics, and microbiological findings of pediatric patients are available in detail in Supplementary Table 1 at https://doi.org/10.6084/m9.figshare.24592368.v1.

### Antimicrobial susceptibility testing

Antimicrobial susceptibility pattern was showed that the highest resistance frequency was for both carbapenems, imipenem, and meropenem (61, 87.1%), followed by cefotaxime (60, 85.7%), ceftazidime, cefepime, ceftriaxone, ciprofloxacin, levofloxacin, amikacin, and cotrimoxazole (59, 84.2%), piperacillin-tazobactam (58, 82.8%), gentamicin (57, 81.4%), tobramycin (56, 80%), tetracycline (44, 62.8%), minocycline (34, 48.5%), ampicillin-sulbactam and doxycycline (33, 47.1%), and tigecycline (3, 4.2%). In total, 61 out of 70 isolates were resistant to both imipenem and meropenem, whereas 8 out of 70 isolates were sensitive to them. However, only one isolate was found to be sensitive to imipenem but was resistant to meropenem.

**Fig 1:**
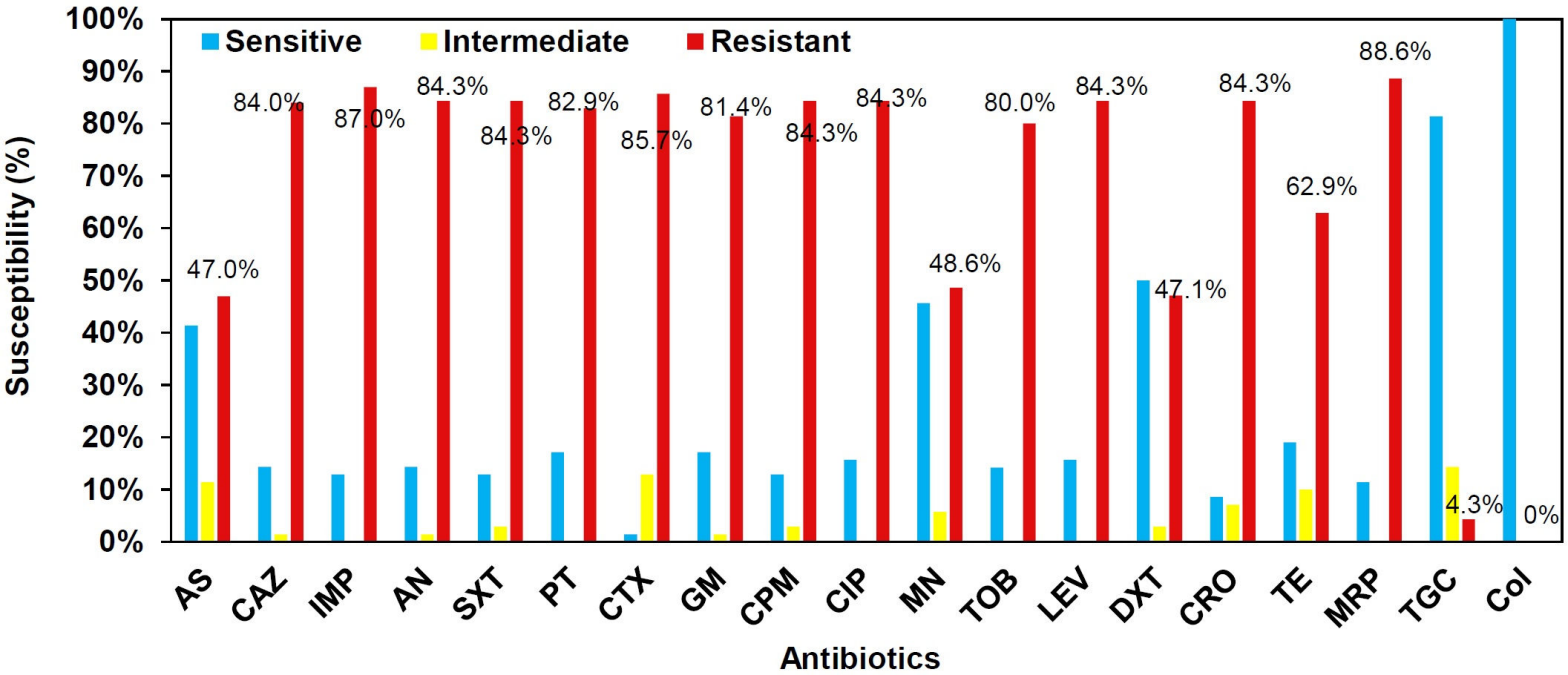
*In vitro* antimicrobial susceptibility patterns of *A. baumannii* clinical isolates. AS, ampicillin-sulbactam; CAZ, ceftazidime; IMP, imipenem; AN, amikacin; SXT, trimethoprim-sulfamethoxazole; PT, piperacillin-tazobactam; CTX, cefotaxime; GM, gentamicin; CPM, cefepime; CIP, ciprofloxacin; MN, minocycline; TOB, tobramycin; LEV, levofloxacin; DXT, doxycycline; CRO, ceftriaxone; TE, tetracycline; MRP, meropenem; TGC, tigecycline; CL, colistin.

All isolates were susceptible to colistin with MICs ranging from 0.03 to 2 µg/mL. The MIC_50_ and MIC_90_ of colistin were 0.5 and 1 µg/mL, respectively. In all, 56 out of 70 (80%) *A*. *baumannii* isolates were categorized as MDR, and 25 out of 70 (35.7%) were XDR. Antimicrobial susceptibility patterns of *A. baumannii* isolates are summarized in Fig. 1.

### Biofilm formation

#### Biofilm formation in the absence of antibiotic

The isolates were divided into groups according to the measurement of OD_570_ nm and OD_NC_ values as previously described above. Hence, 7.1% of the isolates (*n* = 5) were considered non-biofilm producers, and 92.9% of the isolates (*n* = 65) were biofilm producers, among which 22.8% (*n* = 16), 32.8% (*n* = 23), and 37.1% (*n* = 26) of the isolates were grouped as weak, moderate, and strong biofilm producers, respectively. No significant difference was found between the clinical origin of isolates and biofilm formation ability (*X^2^* = 21.596, *df* = 30, *P* = 0.13). The positive control *A. baumannii* ATCC 19606 was categorized as a weak biofilm producer. Moreover, the statistical correlation of biofilm formation and drug resistance phenotypes of *A. baumannii* revealed that 5 out of 56 MDR isolates did not produce biofilm; however, 15 out of 56, 20 out of 56, and 16 out of 56 MDR isolates were weak, moderate, and strong biofilm producers, respectively. All non-MDR isolates (*n* = 14) were biofilm producers, among which 10 non-MDR isolates produced strong biofilm. Spearman’s rank correlation analysis demonstrated that the non-MDR *A. baumannii* tended to form strong biofilm compared to MDR isolates presenting an inverse correlation between the biofilm formation capacity of *A. baumannii* and MDR and XDR phenotypes (rs = 0.355, *P* = 0.003, *n* = 70).

#### Biofilm formation in the presence of antibiotic

A decrease in biofilm formation was observed in the groups of weak, moderate, and strong biofilm producers of *A. baumannii* in the presence of sub-MICs of colistin but no statistically significant correlation was found according to Fisher’s exact test (*P* = 0.057 for ¼ MIC and *P* = 0.085 for ½ MIC).

The biofilm formation ability of *A. baumannii* isolates after exposure to sub-MICs of colistin is shown in Table 3. The association of biofilm formation ability of *A. baumannii* in the absence/presence of sub-MICs of colistin and 18 laboratory-used antibiotics is presented in Supplementary Table 2 at https://doi.org/10.6084/m9.figshare.24592371.v1.

**TABLE 3:** The biofilm formation ability of *A. baumannii* isolates after exposure to sub-MICs of colistin

	Percentage of biofilm formation		
	Colistin free	¼ MIC of colistin	½ MIC of colistin
**Negative**	7%	11%	16%
**Weak**	23%	41%	43%
**Moderate**	33%	30%	27%
**Strong**	37%	17%	14%

### Identification of genes involved in biofilm formation

Molecular analysis of biofilm-related genes in *A. baumannii* isolates displayed that 100% of the clinical isolates (*n* = 70) were positive for the *abaI, ompA*, and *csuE* genes, followed by *bla*_PER-1_ 24.3% (*n* = 17), and *bap* 22.85% (*n* = 16). The genes *abaI, ompA,* and *csuE* were found in both biofilm-producing and non-biofilm-producing isolates. However, *bap* and *bla*_PER-1_ were detected in biofilm producers. The majority of isolates carrying *bla*_PER-1_ were strong biofilm producers. Nonetheless, the *bap* was observed in almost equal frequency in weak, moderate, and strong biofilm producers. We did not observe a significant correlation between the biofilm production capacity and the presence of biofilm-related genes (*P* value > 0.050).

Detailed information about biofilm-associated genes in pediatric isolates of *A. baumannii* is illustrated in Supplementary Table 1 at https://doi.org/10.6084/m9.figshare.24592368.v1.

### Cell adherence and invasion

The six selected biofilm-producing *A. baumannii* isolates were subjected to adherence and invasion assays as listed in Table 4.

**TABLE 4:** The six selected biofilm-producing *A. baumannii* isolates (AB) for adherence and invasion assays

Isolates	MIC of colistin (μg/ml)	Colistin free	¼ MIC of colistin	½ MIC of colistin	MDR/XDR
AB-7	0.5	Strong	Strong	Strong	**–**
AB-32	0.5	Strong	Moderate	Moderate	MDR
AB-35	2	Weak	Moderate	Moderate	XDR
AB-36	1	Strong	Weak	Weak	**–**
AB-37	1	Strong	Strong	Moderate	**–**
AB-59	0.25	Strong	Weak	Weak	MDR

The adhesion of selected AB isolates to the HEp-2 cells was diminished, except for AB-36, at ½ MICs of colistin compared to their adhesion in the absence of colistin. The adhesion of AB-7, AB-32, AB-35, and AB-59 isolates to the HEp-2 cells was decreased at ¼ MICs of colistin when compared to their adhesion in the absence of colistin. In addition, the adhesion of AB-36 and AB-37 isolates to the HEp-2 cells was enhanced at ¼ MICs of colistin.

**Fig 2:**
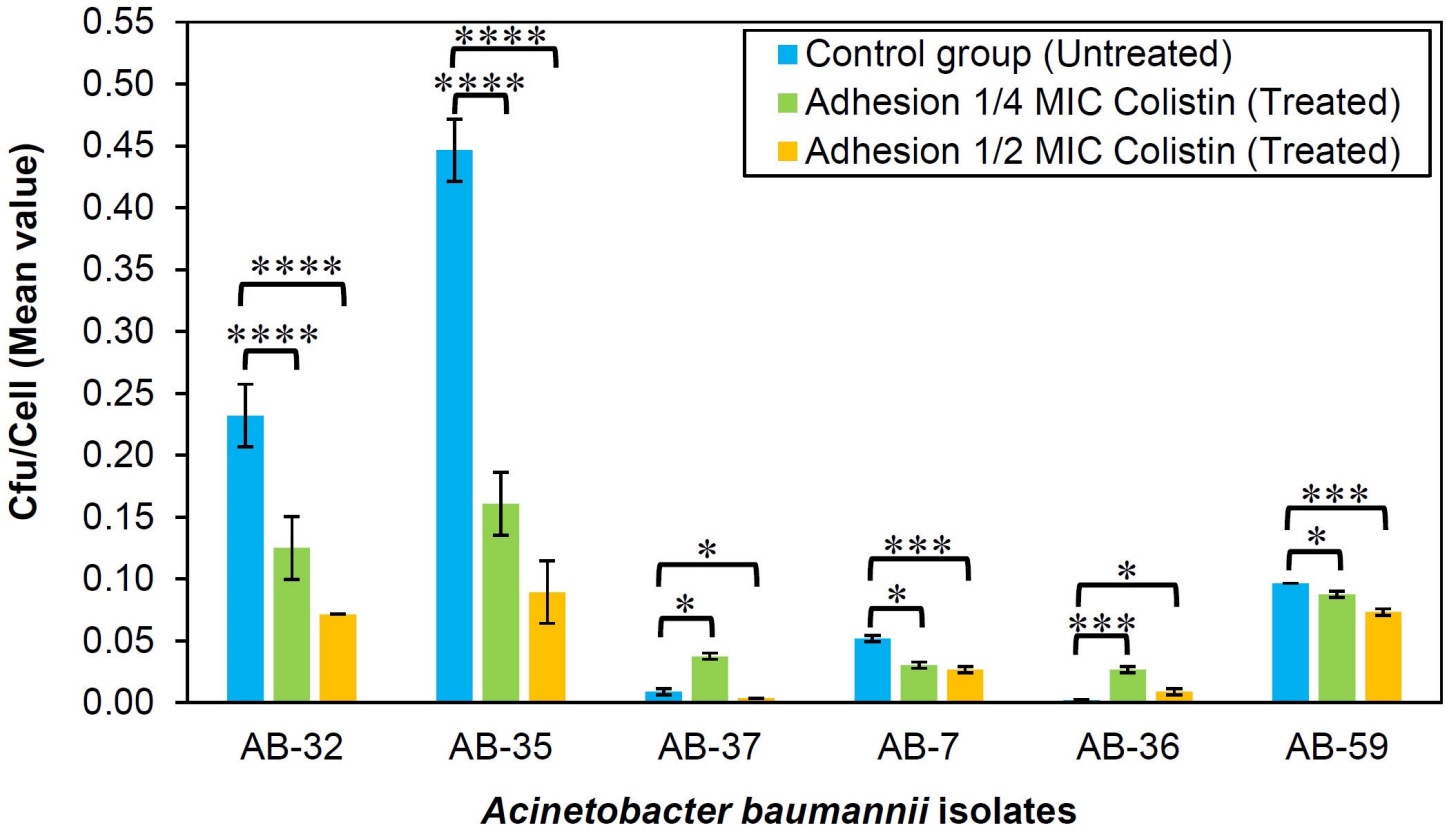
Adhesion of *A. baumannii* isolates to the HEp-2 cells at 0 MIC (Untreated), ¼ MIC, and ½ MIC of colistin. Adhesion ratio: the number of bacteria attached to HEp-2 cells/the number of cells on the surface of a 24-well polystyrene plate compared to untreated conditions that did not contain colistin. The asterisks *, ***, and **** indicate a statistically significant difference (*P* value > 0.0316, *P* value > 0.0016, and *P* value > 0.0001, respectively) compared to the control group. Error bars were assigned based on the standard deviation (SD).

However, a significant correlation was observed in the adhesion of AB isolates to the host cells at ½ and ¼ MICs of colistin when compared to the control group (0 MIC) as displayed in Fig. 2. By contrast, none of the AB isolates was able to invade the HEp-2 cells in both absence and presence of sub-MICs (½ and ¼ MICs) of colistin.

### Quantitative real-time PCR

The quantitative real-time PCR (qPCR) analysis of mRNA expression levels of *csuE* and *ompA* in the biofilm-forming *A. baumannii* clinical isolates following treatment with sub-MICs of colistin revealed that the mRNA levels of *csuE* and *ompA* were decreased in AB-7, AB-32, and AB-35 isolates in the presence of ½ and ¼ MICs of colistin compared to the absence of colistin. The mRNA levels of *ompA* were decreased at ½ MICs, and also for *csuE* at ½ and ¼ MICs of colistin in comparison with the absence of colistin in AB-36. In addition, a decrease in the mRNA levels of *csuE* and *ompA* was observed at ½ MICs when compared to the absence of colistin, while the mRNA levels of *csuE* were enhanced at ¼ MICs of colistin in AB-37. On the other hand, no changes were observed in the mRNA levels of *ompA* at ¼ MICs in AB-35 and AB-37, as well as for AB-59 at ½ and ¼ MICs in comparison with the absence of colistin. However, a significant increase was seen in the mRNA levels of *csuE* at ½ and ¼ MICs when compared to the absence of colistin. In AB-36, the mRNA levels of *ompA* were also increased at ½ MICs compared to the absence of colistin. Spearman’s rank analysis disclosed a weak positive significant correlation between the biofilm formation and expression levels of *csuE* and *ompA* at ½ and ¼ MICs of colistin (rs = 0.153); by contrast, a strong positive significant correlation was found between the biofilm formation and expression levels of the biofilm-related genes in the absence of colistin (rs = 0.851).

**Fig 3:**
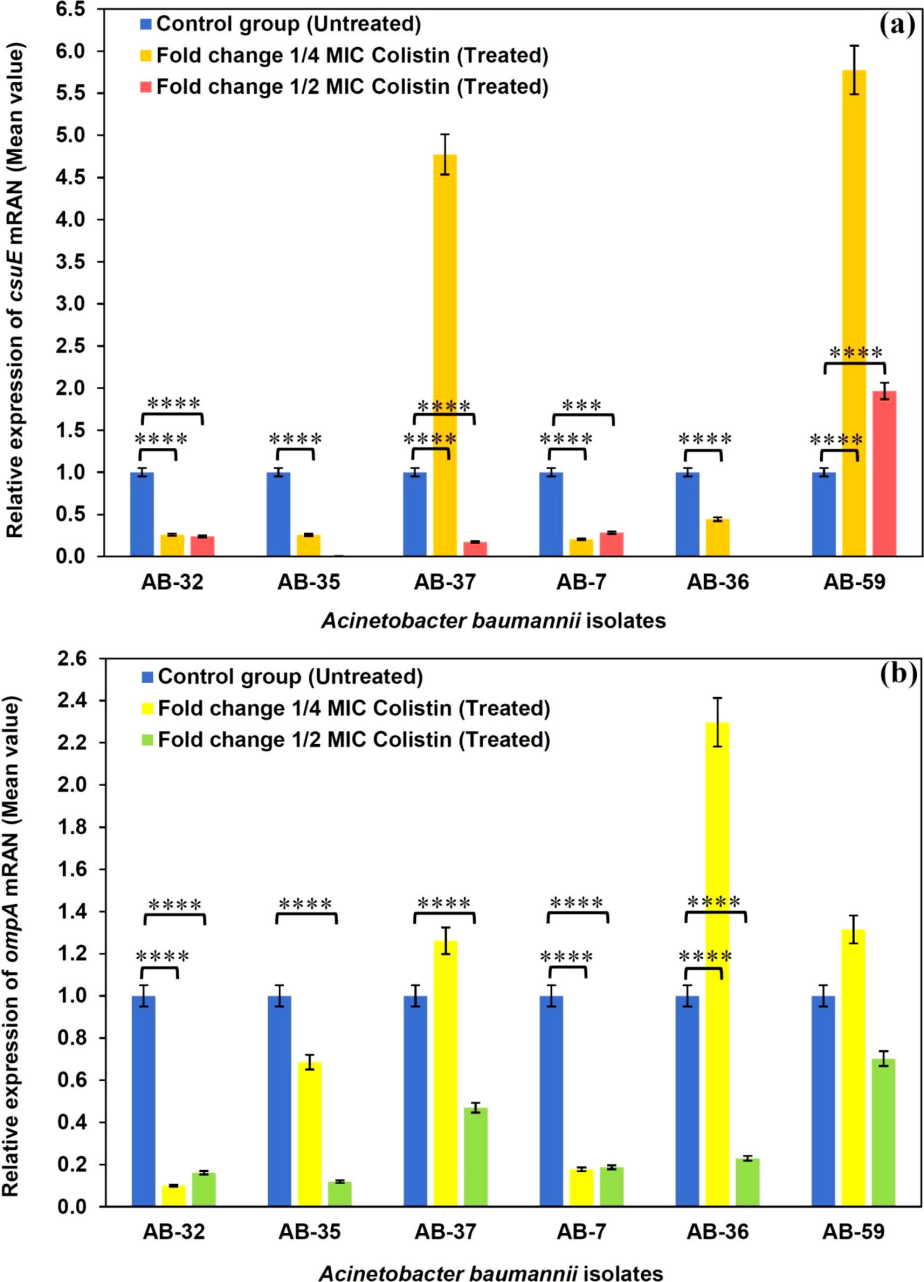
Statistical analysis of the qPCR results on the expression levels of *csuE* (a) and *ompA* (b) in biofilm-forming *A. baumannii* clinical isolates at 0 MIC (untreated), ¼ and ½ MICs of colistin. The bar graph data are shown as the mean ± SEM (*n* = 6) of 3 independent experiments. The asterisks indicate statistically significant differences between the untreated bacteria and antibiotic-treated bacteria.

Statistical analysis of the qPCR results on the expression levels of *csuE* and *ompA* in biofilm-forming *A. baumannii* clinical isolates at ½ and ¼ MICs of colistin are shown in Fig. 3.

## DISCUSSION

In this study, we attempted to understand sub-MICs of colistin downregulating biofilm-associated genes as well as weakening biofilm formation and adherence of *A. baumannii* to the host cells to make an insight into current gaps in combination therapy, specifically in biofilm-related infections and multiple drug-resistant strains in children that need to be addressed before realistic treatment failure mitigation strategies can be developed.

Here, we focused on colistin as the “last-resort” antibiotic for the treatment of drug-resistant *A. baumannii* infections. Since the toxicity of colistin is dose dependent (36), there is a focus on strategies that reduce the dose while maintaining the therapeutic effect of the drug (37). Hence, the sub-MICs of colistin were addressed for the growth, biofilm formation, adherence, and invasion capacities of *A. baumannii* clinical isolates. We found no significant difference between the clinical origin of *A. baumannii* isolates and biofilm formation ability, as also described by Bogdan et al (38).

*In vitro* biofilm formation assay using a microtiter-plate test showed that 93% of the isolates were biofilm producers, among which almost half of the isolates were able to form a strong biofilm, which is in concordance with previous reports (39–41). Up to now, several studies have been conducted to find out the relationship between the ability of biofilm formation and antimicrobial resistance in bacteria (42, 43). In our study, there was no statistically significant difference between the biofilm formation and *A. baumannii*-resistant strains to either 18 laboratory-used antibiotics, except for piperacillin-tazobactam, ciprofloxacin, tobramycin, levofloxacin, ceftriaxone, tetracycline, and tigecycline at colistin free; amikacin and ceftriaxone at ¼ MIC of colistin; minocycline, for ½ MIC of colistin. Therefore, it is possible that the small sample sizes in some groups could have masked significant differences in those groups. Some previous studies have shown that a negative relationship exists between the biofilm formation capacity and MDR/XDR phenotypes of *A. baumannii* (44, 45), corresponding with our report. Overall, the negative correlation between the biofilm formation and multiple resistance in *A. baumannii* has less been reported in comparison with the positive correlation studies. Currently, the correlation between drug resistance and biofilm formation in microorganisms is a challenging issue that may be affected by the origin and distribution of phylogenetic groups (46).

It has been demonstrated that the biofilm-related virulence determinants, including *bap*, *abaI*, *ompA*, *csuE*, and *bla*_PER-1_ are associated with multidrug resistance in *A. baumannii* (47).

The distribution of biofilm-associated genes presented a high-frequency rate (100%) for *ompA*, *csuE*, and *abaI*, followed by *bap* and *bla*_PER-1_ genes (22.85% and 24.3%, respectively), which is in agreement with previous studies (40, 41). However, the presence of the virulence genes did not show a significant correlation with biofilm formation. It has been reported that most *A. baumannii* strains carrying the *bap* gene can form biofilm on both biotic and abiotic surfaces, supporting the theory that *bap*, as a key factor, may be involved in bacterial adhesion (48). The *bla*_PER-1_ was found in the highest frequency in strong biofilm producers of *A. baumannii* strains in our study, although a statistically significant correlation was not achieved. A study also showed a weak significant correlation between the presence of *bla*_PER-1_ and biofilm formation (49). By contrast, a strong significant correlation between the two variables has been found in other studies. It has been recognized that *A. baumannii* strains carrying the resistance determinant *bla*_PER-1_ can form a strong biofilm compared to strains without *bla*_PER-1_ (50, 51). It seems that *bla*_PER-1_ enhances the adhesion of organisms carrying the gene without necessarily contributing to biofilm formation (40). The *ompA* was detected in both biofilm producers and non-biofilm producers of *A. baumannii* in our study, which is in concordance with previous studies (40). However, further evidence is not available to specify whether *OmpA* induces biofilm formation. A key role of *csuE*, a member of the chaperone-usher secretion machinery, in the biofilm formation of *A. baumannii* has also been explored in several studies (52, 53).

A possible reason for the lack of significant correlation between the biofilm formation capacity and biofilm-related genes is that other genetic determinants/regulators may be involved in biofilm formation. In addition, mutations may occur in different regulatory systems affecting the expression and/or function of biofilm-related proteins, as described by a previous study (54). As well as, the effect of drug classes can even vary from one strain to another, especially when an antibiotic has a dual effect against different species *in vivo*. Therefore, effective concentrations of antibiotics may reduce biofilm formation in a particular clone and simultaneously induce another one (55). Further studies are needed to understand the clinical aspects of molecular mechanisms of biofilm formation.

The mechanisms behind sub-MIC antibiotic-induced biofilm formation have less been studied.

It has been said that sub-MICs of some antibiotics are unable to kill bacteria but can inhibit biofilm formation (19). By contrast, several studies have shown that sub-inhibitory doses of antibiotics can significantly induce biofilm formation in a variety of bacterial species *in vitro* (54, 56). It has been suggested that biofilm formation is not directed by a single factor or a specific genotype and it is an exceedingly complicated biological phenomenon that is regulated by several effectors and seems to be strain dependent (53). Our findings showed that sub-inhibitory concentrations of colistin changed the expression levels of genes encoding biofilm-related proteins in *A. baumannii*. A positive significant correlation between the expression levels of biofilm-related proteins and sub-MICs of antibiotics has been reported previously (23, 57, 58). Another study represented that exposure of *A. baumannii* at sub-MICs of imipenem and colistin did not significantly modify the expression of the *csu* operon (22). Recently, the transcriptional analysis revealed that bacterial stress responses against sub-lethal doses of antibiotics are broad ranged in comparison with specific responses; thus, the expression of virulence factors may be subsequently suppressed (59, 60).

Little is known about the adhesion and invasion of *A. baumannii* as well as the interaction of organisms with host cells contributing to pathogenicity. We displayed that the sub-MICs of colistin could detract adhesion of *A. baumannii* isolates, while none of the isolates were able to invade the HEp-2 host cells in both the absence and presence of sub-MICs of colistin, which is consistent with the previous study (61). The sub-inhibitory antibiotic-induced adhesion and sub-MIC antibiotic-inhibited adhesion of *A. baumannii* strains have been differently reported in previous studies (62–64). Some studies highlight that *A. baumannii* infection can lead to apoptosis without cell invasion (53, 65). Furthermore, the incubation time longer than 5 hours leads to a decrease in the viability of eukaryotic host cells, as described by a previous study (65). According to the findings, we believe *A. baumannii* strains have little tendency to invade host cells. However, a relatively low ability of *A. baumannii* to adhere and invade host cells may contribute to the low virulence of this opportunistic pathogen, particularly in critically ill patients and children (66, 67). Further work is needed to clarify the underlying mechanisms of antibiotics at sub-MICs on genes conferring resistance and expression of genes encoding important virulence factors involved in the biofilm formation, adhesion, and pathogenicity of *A. baumannii*.

There were a number of limitations to the current study: (i) our sample size was relatively small, (ii) the present study was limited to a single-center experience, (iii) our research was limited to clinical samples of *A. baumannii* and environmental samples of *A. baumannii* were not studied, (iv) the other important genes that may be involved in biofilm formation were not examined, and v) the sub-MICs of other effective therapeutic antibiotics were not included in this research.

### Conclusion

Our study did not show a significant association between the biofilm-forming capacity and antibiotic resistance phenotypes in *A. baumannii* clinical isolates. Considerable diversity in biofilm formation ability and prevalence of biofilm-specific genes was also observed among *A. baumannii* isolates. Our findings showed that the genes encoding biofilm-associated proteins changes expression levels following sub-MICs of colistin in *A. baumannii* clinical isolates. Although our data were obtained from *in vitro* studies and cannot be attributed to *in vivo*, they provide insight into the mechanisms of bacterial response to sub-MICs of antibiotics and lay the foundation for future *in vivo* studies. These findings provide new insight for pediatricians and stimulate interest in the development of therapeutic strategies for ESKAPE pathogens.

## Data Availability

The nucleotide sequences of *csuE*, *bap*, and *bla*_PER-1_ were deposited in GenBank under accession no. ON037456, OM471871, and ON037455, respectively.
